# Standing on unstable surface challenges postural control of tracking tasks and modulates neuromuscular adjustments specific to task complexity

**DOI:** 10.1038/s41598-021-84899-y

**Published:** 2021-03-17

**Authors:** Lida Mademli, Dimitra Mavridi, Sebastian Bohm, Dimitrios A. Patikas, Alessandro Santuz, Adamantios Arampatzis

**Affiliations:** 1 School of Physical Education and Sport Science at Serres, Aristotle University of Thessaloniki, Agios Ioannis, 62110, Serres, Greece; 2 School of Medicine, Aristotle University of Thessaloniki, Thessaloniki, Greece; 3 School of Physical Education and Sport Science, Aristotle University of Thessaloniki, Thessaloniki, Greece; 4 Department of Training and Movement Sciences, Humboldt-Universität zu Berlin, Philippstr. 13, Haus 11, 10115 Berlin, Germany; 5 Berlin School of Movement Science, Humboldt-Universität zu Berlin, Berlin, Germany

**Keywords:** Motor control, Central pattern generators

## Abstract

Understanding the modulations of motor control in the presence of perturbations in task conditions of varying complexity is a key element towards the design of effective perturbation-based balance exercise programs. In this study we investigated the effect of mechanical perturbations, induced by an unstable surface, on muscle activation and visuo-postural coupling, when actively tracking target motion cues of different complexity. Four postural tasks following a visual oscillating target of varying target complexity (periodic-sinusoidal vs. chaotic-Lorenz) and surface (stable-floor vs. unstable-foam) were performed. The electromyographic activity of the main plantarflexor and dorsiflexor muscles was captured. The coupling between sway and target was assessed through spectral analysis and the system’s local dynamic stability through the short-term maximum Lyapunov exponent. We found that external perturbations increased local instability and deteriorated visuo-motor coupling. Visuo-motor deterioration was greater for the chaotic target, implying that the effect of the induced perturbations depends on target complexity. There was a modulation of the neuromotor system towards amplification of muscle activity and coactivation to compensate surface-related perturbations and to ensure robust motor control. Our findings provide evidence that, in the presence of perturbations, target complexity induces specific modulations in the neuromotor system while controlling balance and posture.

## Introduction

There is evidence that the presence of noise in the nervous system, i.e. random or unpredictable fluctuations and disturbances in neural information processing from the perception of sensory signals to the generation of motor commands^1^, facilitates the ability of the motor system to detect and transmit sub-threshold sensory signals^2,3^. Furthermore, neural networks that are formed in the presence of noise may be more efficient to cope with environmental changes than those in low levels of noise^1,3^. External mechanical perturbations (i.e. alteration of the function of a biological system induced by external mechanisms) enhance the demand of the sensorimotor transformation and increase muscle activation^4–8^. Both the increase of muscle activation level and the enhancement of sensory inputs due to extrinsic disturbances are biologically relevant sources of noise that may contribute to stochastic facilitation^9^. Current studies^7,10^ report that the motor system in the presence of perturbations increases robustness of motor control (i.e. the ability to cope with errors of execution) to create safe conditions and that proprioception shows a relevant contribution to robustness modulation^11^.

In the presence of external mechanical perturbations, the local dynamic stability during walking^10,12,13^, running^10^ and balance tasks^7,14^ decreases. At the same time, electromyographic (EMG) activity and muscle coactivation increases^14–16^, indicating a modulation of the neuromotor control in the presence of perturbations. Furthermore, during perturbed conditions the basic activation patterns of muscle groups become wider increasing the temporal overlap of activation between chronologically adjacent muscle synergies^10,17,18^. Similar modifications in motor control have been observed in wild-type mice but not in genetically modified mice that lack feedback from proprioceptors^11^, showing the relevant contribution of sensory information in the modulation of motor control. Recent studies^17,19,20^ reported a reduction in the complexity of motor control in demanding conditions in order to ensure robust locomotion. The above reports demonstrate that, although perturbations might let balance performance decrease, the neuromotor system modulates motor control and increases control robustness, possibly an efficient way of counteracting perturbations and preventing a fall.

It seems that perturbations increase the demand of the sensorimotor integration and force the central nervous system to trigger specific modulations, in order to cope with the demanding conditions^7^. One common method to induce external mechanical perturbations and to increase the demand of postural control is the use of unstable surfaces such as foam^21–23^. Unstable conditions during visually guided postural tasks may challenge the neuromotor system to maintain stability and may initiate specific modifications in motor control in order to counteract or compensate for the external induced perturbations. The complexity of the target motion is an additional condition influencing the visuo-motor coupling performance^24,25^. A target moving in an unpredictable, more complex way (chaotic) than a predictable, less complex one (sinusoidal), deteriorates postural sway and target motion coherence^24,25^ evidencing the effects of target complexity on the visuo-motor performance. Oullier et al.^26^ using the moving room paradigm found a decrease in the room-head coupling with increasing room frequency depicting a demand-dependent deterioration of visual coupling performance. While the effect of unstable surfaces on postural control during quiet stance has been widely investigated^14,21,27,28^, there is still a gap in the literature regarding possible modulations of motor control in the presence of perturbations during more complex tasks, such as visually guided postural sway, especially when the target is moving with high complexity.

The present study aimed to investigate the effect of perturbations, induced by an unstable surface, on muscle activation and visuo-postural coupling of sway, when actively tracking either periodic or more complex (chaotic) visual target cues. We hypothesised that the unstable surface would increase the instability of the system and deteriorate the visuo-motor coupling between target and centre of pressure (CoP), yet initiating neuromuscular responses in order to deal with the induced perturbations. Furthermore, we hypothesised that the deterioration of the visuo-motor coupling would be greater when tracking the more complex target.

## Methods

### Participants

A statistical power analysis in G*Power (3.1.9.2, HUU, Düsseldorf, Germany) was performed for the necessary sample size. We estimated the effect size f = 0.49 based on data of previous studies^14^, for examining the surface effect (stable vs unstable) on the coherence value. For a power of 0.9 in a within repeated measures analysis of variance (ANOVA) (one group, two measurements, alpha = 0.05, nonsphericity correction = 1, correlation = 0.5) a sample size of 13 participants would be sufficient to achieve the desired statistical power of the expected outcome. To account for a potential drop-out, we recruited twenty healthy adults, with no history of neuromuscular or musculoskeletal disorders, to participate in the study (12 male and 8 female, height 175 ± 7 cm, body mass 69 ± 12 kg, age 32 ± 5 years, mean ± SD). From seven participants not all data (kinematic, EMG, force plate) were accurately collected for all examined trials, thus the data from thirteen adults were used in the analysis (8 males and 5 females, height 176 ± 1 cm, body mass 71.8 ± 11.1 kg, age 33 ± 5 years, mean ± standard deviation). All participants were informed about the experimental protocol and gave their informed consent before their inclusion to the study. The experiment was performed with the approval of the institution's ethics committee (HU-KSBF-EK_2018_0013, Humboldt-Universität zu Berlin) and in accordance to the Declaration of Helsinki.

### Task and apparatus

**Figure 1:**
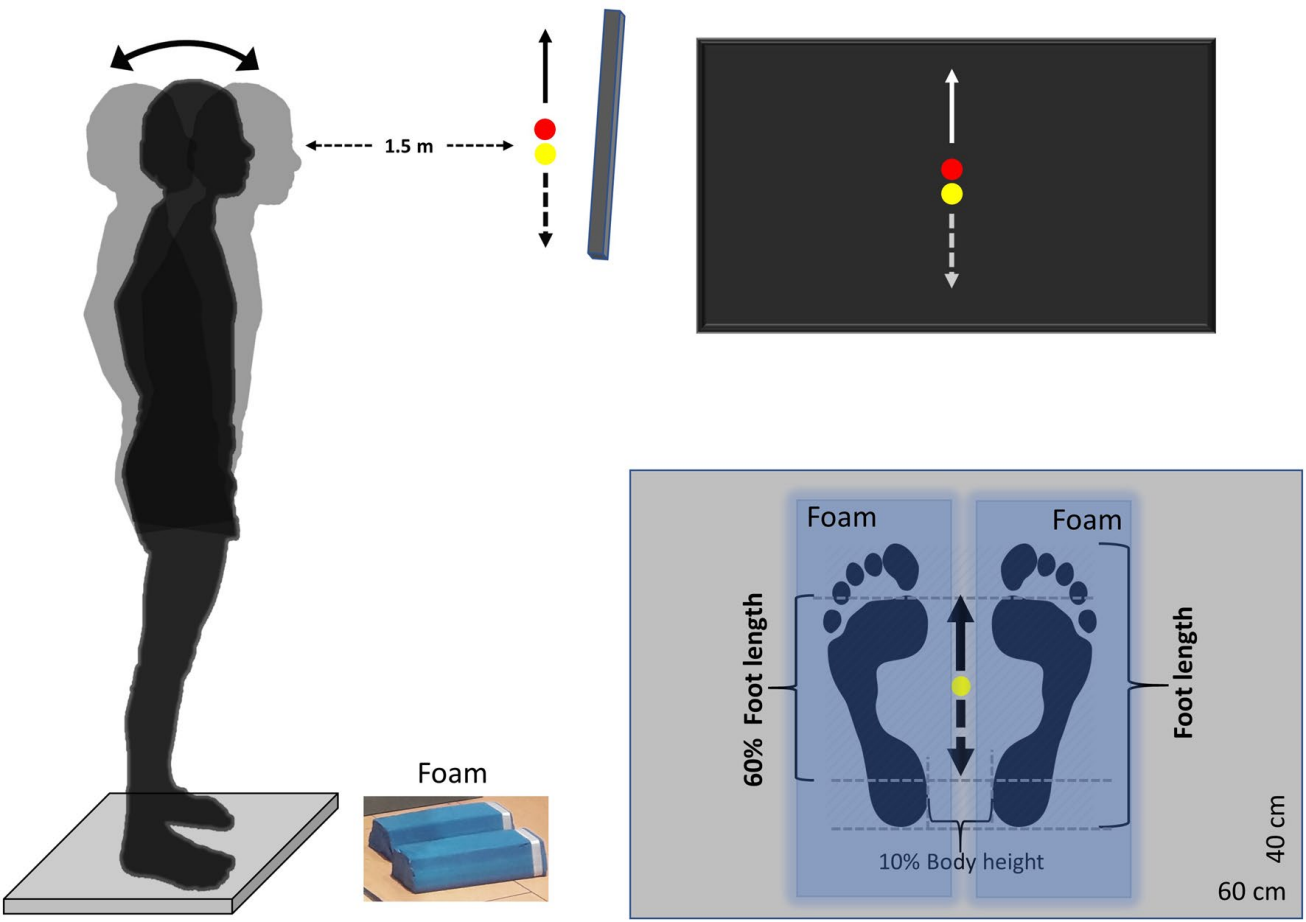
Experimental set up. Participants had to track the visual target (red dot) moving in the vertical direction with the yellow dot, whose motion was controlled by the motion of their centre of pressure (CoP) along the anteroposterior direction. The participants had to keep their feet on the ground and lean their body in the anterior–posterior direction. An anterior CoP shift corresponded to an upward dot motion (continuous arrow line), while a posterior shift to a downward dot motion (dashed arrow line). The target’s motion amplitude was normalised to 60% of the participant’s foot length. Each tracking task lasted 120 s. Overall, four postural tasks were performed, which varied in target complexity (periodic-sinusoidal vs. chaotic-Lorenz signal target) and surface (stable-rigid vs. unstable-foam surface). Areas shaded in blue designates the position of the foam pads for the trials on unstable ground.

Participants were asked to perform four different postural tasks, consisting in following a visual vertically oscillating target, which was displayed on a large TV screen (119.38 cm, HD LG), by shifting their weight along the anteroposterior direction (Fig. 1). As in previous studies^14,25^ the target had the shape of a dot which moved in the middle of the screen in a vertical direction. Upwards motion of the dot corresponded to anterior direction of the CoP, while downwards motion to posterior CoP direction. The four postural tasks varied in target complexity (periodic-sinusoidal vs. chaotic-Lorenz signal target) and surface (stable-floor vs. unstable-foam): (a) tracking a sinusoidal target on rigid surface, (b) tracking a chaotic-Lorenz target on rigid surface, (c) tracking a sinusoidal target on foam surface and (d) tracking a chaotic-Lorenz target on foam surface. The foam surface consisted of two balance beams (Sport-Thieme Balance beam EVA foam, 38 × 16.5 × 5.8 cm, Sport-Thieme Germany).

Participants were asked to stand barefoot on a force platform (40 × 60 cm, AMTI BP400600-2000, Advanced Mechanical Technology, Inc., Watertown, MA, USA) having the arms akimbo and feet placed in the middle of the platform with inter-malleolar distance at 10% of body height. The hands were positioned at the level of pelvis to avoid any contribution/assistance of arms’ motion in swaying that could have influenced task performance, in order to control for confounding factors. On the screen two different coloured dots were shown: the target in red, and a yellow one, depicting the participant’s anteroposterior CoP component, serving as the instantaneous performance feedback signal. The instruction given to the participants was to follow the motion of the red dot, by controlling the motion of the yellow one as they shifted their weight anteroposteriorly, and to keep their knees and hip straight. The duration of the tracking trials was 120 s. Each participant performed one trial for each condition, in a randomised sequence to avoid possible order effects. Participants were provided ample time (about 30 s) to familiarise with the task before the actual testing trial. Prior to each trial, room lights were dimmed.

The participants’ postural behaviour was recorded using the force platform at 1,000 Hz, a motion capture system (Vicon, Oxford, U.K.) and a wireless electromyography system device (myon m320, myon AG, Schwarzenberg, Switzerland). Ten infrared-cameras captured the kinematic data of sixteen reflective markers (diameter 14 mm) at a sampling rate of 250 Hz; on lateral malleolus, medial and lateral epicondyle of the femur and greater trochanter bilaterally, as well as the 2nd, 7th 10th thoracic vertebra, 2nd lumbar vertebra, and 4 on the head placed on a headband: two on the front (left and right) and two on the back (left and right). The electromyographic activity of soleus (SOL), gastrocnemius medialis (GM), gastrocnemius lateralis (GL) and tibialis anterior (TA) was recorded at a sampling frequency of 1,000 Hz. These muscles were recorded, because they have been reported to play an important role in postural control, as they oppose the destabilizing effects of gravity^29^. The wireless EMG electrodes were placed at the right lower limb, on the belly of each muscle, with 2 cm intra-electrode distance according to SENIAM recommendations. Force, kinematic and EMG signals were simultaneously recorded and stored for further processing.

### Target motion

The target’s motion was constructed in MATLAB (version R2014b, MathWorks Inc., USA) using two signals of different complexity, one periodic and one chaotic. In both of them, the maximum (peak to peak) amplitude of target motion was set to 60% of the foot length, which corresponded to an average length of 15.02 ± 1.19 cm (Fig. 1). This amplitude was selected after pilot tests, which revealed that 60% of the foot length is an appropriate amplitude for swaying on stable and on unstable surfaces, since it induces an increase in the instability on the unstable surface compared to the rigid one, similarly to previous studies^7,10^. The periodic signal was a sinewave with a single frequency (f) set at 0.25 Hz that was generated using the sin function $[sine(t)=\mathrm{sin}\left(2\pi i\times \mathrm{f}\times t\right)]$. The particular frequency was selected because this is the dominant frequency of natural, self-paced voluntary sway^30,31^. The chaotic signal was derived from a Lorenz attractor, according to the parameters: σ = 10, β = 8/3 and r = 28 and the initial conditions: x_0_ = 0.1, y_0_ = 0.1 and z_0_ = 0.1. The signal characteristics were: h (time resolution) = 0.004, steps (number of points) = 10,000 (we chose 6,000 data points from y-axis [y (4,000: 10,000)]), noise flag = 0^24,32^. The frequency range for the Lorenz signal was confined between 0 and 1 Hz, which is an ecologically valid spectrum of frequencies for voluntary sway^33^ and has a power spectrum with one dominant frequency around 0.25 Hz. Both target and CoP anteroposterior position feedback were synchronously shown on the TV monitor by means of a custom-built software (developed in MATLAB) with 50 Hz refresh rate (6,000 data points for each signal during 120 s).

### Data analysis

#### Spectral analysis

Spectral analysis in the frequency band between 0 and 1 Hz was used for assessing the CoP-target motion coupling, based on the methods of Halliday et al.^34^ by means of a customised version of a freely available software (NeuroSpec 2.0). Both the CoP and target time series were interpolated at 64 Hz in order to achieve an appropriate frequency resolution for the spectral analysis, as we wanted to get a coherence value at 0.25 Hz (frequency of the sinewave) and the neighbour frequencies (for the Lorenz signal). The spectral coherence was used as a metric of correlation between the two signals (CoP-target position) in the frequency domain, illustrating their linear relationship.

#### Local dynamic stability assessment

The local dynamic stability was assessed through the short-term maximum Lyapunov exponents (sMLE), which quantifies the rate of divergence of nearby trajectories in state space^35,36^. The analysis followed the procedure used in a previous study^37^. The anatomical landmarks were represented by the 3D coordinates of the markers as follows: (a) ankle—lateral malleolus marker; (b) knee—by clustering lateral and medial epicondyle femur marker; (c) hip—trochanter major; (d) spine—by clustering of the 2nd, 7th,10th thoracic and 2nd lumbar vertebrae; (e) head—by clustering the four head markers. The sMLE was calculated on the norm of the 3D trajectories of the ankle, knee, hip, spine and head landmarks. Initially, the original time series have been filtered using a 4th order Butterworth zero-lag low-pass filter with a cut-off frequency of 20 Hz and consequently down-sampled to 20,000 data points. To reconstruct the state space from the one-dimensional time series, we used delay-coordinate embedding^38^ as follows:

1 $$S\left(t\right)=\left[z\left(t\right),z\left(t+\tau \right),\dots ,z(t+\left(m-1\right)\tau )\right]$$

with S(t) being the m-dimensional reconstructed state vector, z(t) the input 1D coordinate series, τ the time delay and m the embedding dimension. Time delays were selected based on the first minimum of the Average Mutual Information function^39^. For these data, m = 3 was sufficient to perform the reconstruction. Individually selected time delays were chosen by averaging the outcome delays deriving from both trials performed by the participants^40^, with τ ranging from ~ 0.20 to ~ 0.27 of the overall cycle. Further, the average divergence of each point’s trajectory to its closest neighbour was calculated, using the Rosenstein algorithm^41^. The sMLE value was calculated as the slope of the average divergence curves’ linear fit corresponding to the individuals’ delay value at the 0.25 of the average postural sway cycle (i.e., the most linear part of the curve).

#### EMG processing

EMG activity was processed and analysed in MATLAB (R2014b). The EMG signals were band-pass filtered between 10 and 450 Hz, full-wave rectified and then smoothed with a low-pass filter at 5 Hz; a 4th order Butterworth zero-lag filter was used for both. For each participant and each muscle, the average EMG activity of all four investigated trials was calculated for the given muscle. This value was used as the constant reference for the normalisation of the EMG signal for each corresponding participant and muscle, in all trials. In order to remove the baseline activation of each muscle and for each participant, the minimum value of the normalised EMG signal from all four trials was subtracted from the normalised EMG signal^14^. The EMG activity of the triceps surae muscle (TS) was calculated at each time point as [TS_EMG_ = (GM_EMG_ + GL_EMG_ + SOL_EMG_)/3] and it was used as an indicator for the activation of all three triceps surae muscles to assess the coactivation between plantarflexor (expressed by the EMG activity of the TS) and dorsiflexor (TA) muscles during the examined tasks. Then, the root-mean-square (RMS) of the EMG signals throughout the whole trial was calculated, for each muscle and muscle group (GM_RMS_, GL_RMS_, SOL_RMS_, TS_RMS_ and TA_RMS_, respectively). The ratio between TA_RMS_ and TS_RMS_ was calculated as indicator for muscle coactivation during the tasks.

#### Analysed parameters

For task performance assessment, the coherence values were used with values ranging between 0 and 1, and 1 indicating a perfect CoP-target coupling. The sMLE was calculated to quantify the effect of the unstable surface on the local dynamic stability, with higher values demonstrating higher local instability. The RMS of the EMG signal and the coactivation ratios were calculated to assess the modulation of the neuromuscular control in the presence of perturbations.

### Statistical analysis

A repeated measures two-way ANOVA was used with surface type and target complexity as within-subject factors, to examine the effect of surface (stable-floor vs. unstable-foam) and target complexity (periodic-sinusoidal vs. chaotic-Lorenz) on all analysed parameters. For all tests the level of significance was set to α = 0.05. In case of a significant interaction between surface type and target complexity, we performed *post-hoc* comparisons between the surface conditions separately on each target motion using dependent samples t-test, considering the Benjamini–Hochberg correction^42^. The normal distribution of the parameters (for the analysis of variance) and the corresponding differences (for the t-tests) was checked with the Shapiro–Wilk test. All measures were normally distributed.

## Results

### Local dynamic stability

The sMLE of the kinematic parameters was significantly greater when performing the sway on the unstable compared to the stable surface; ankle (F = 10.095, p = 0.006), knee (F = 66.274, p < 0.001), hip (F = 27.466, p < 0.001), spine (F = 17.629, p = 0.001) and head marker (F = 20.080, p < 0.001), indicating that the unstable surface increased local instability in the investigated tasks (Table 1). There was no target complexity (periodic vs. chaotic) effect on the sMLE for all kinematic parameters.

**Table 1:** Short-term maximum Lyapunov exponents of the four examined postural tasks of varying surface (stable-rigid and unstable-foam) and target complexity (periodic-sinusoidal and chaotic-Lorenz) for every analysed anatomical landmark.

Anatomical landmarks	Stable periodic	Unstable periodic	Stable chaotic	Unstable chaotic
Head*	9.25 ± 2.25	10.88 ± 2.60	10.29 ± 1.74	11.41 ± 2.13
Spine*	9.75 ± 1.63	11.14 ± 1.35	9.93 ± 1.10	10.9 ± 2.03
Hip*	9.56 ± 0.98	10.75 ± 1.16	10.18 ± 1.48	11.32 ± 1.98
Knee*	9.31 ± 1.10	11.22 ± 1.10	10.25 ± 1.64	11.52 ± 1.74
Ankle*	9.41 ± 1.21	10.00 ± 1.43	10.20 ± 1.77	11.05 ± 2.06

Values are presented in mean ± standard deviation. *Significant surface effect (stable vs. unstable surface) (p < 0.05).

### Spectral analysis

**Figure 2:**
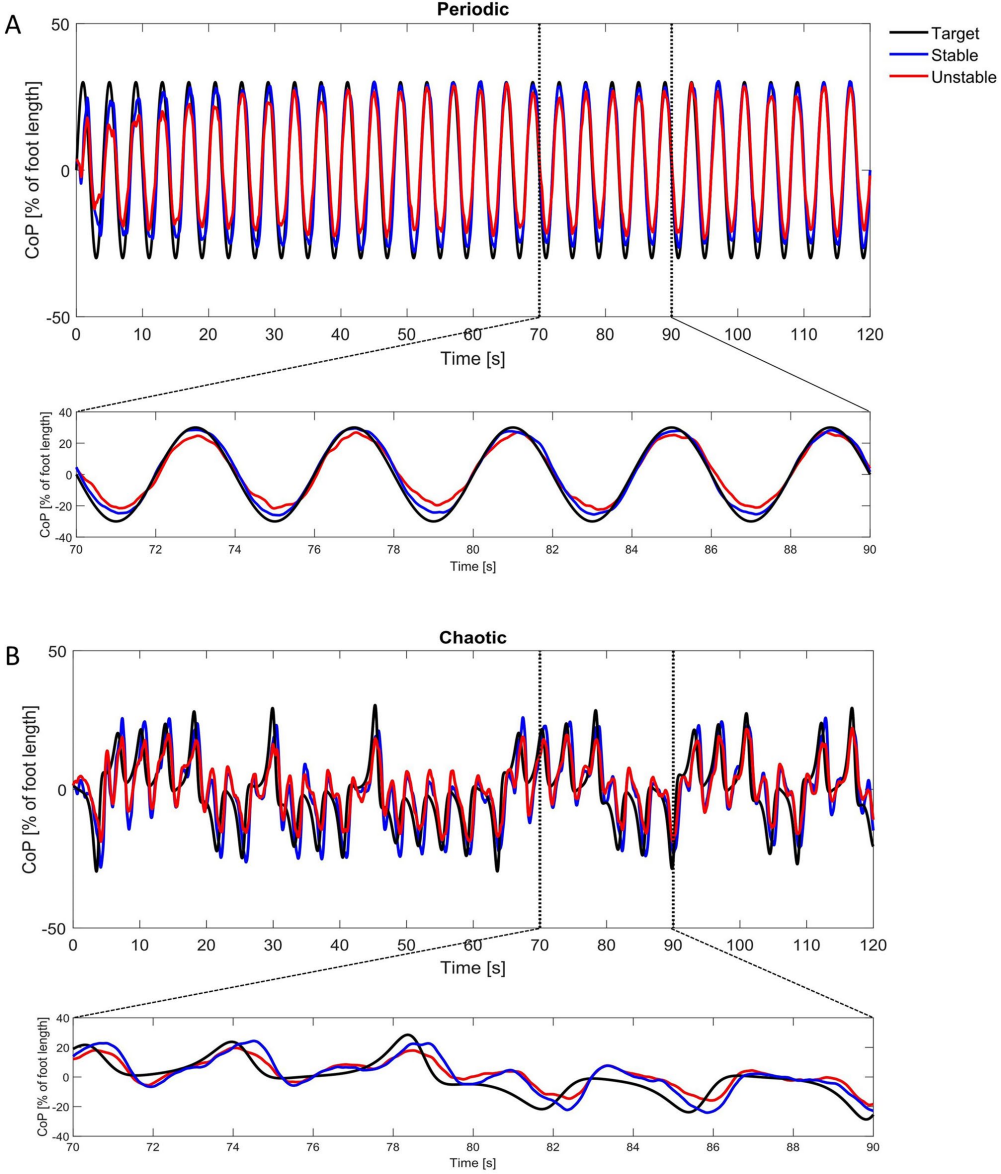
Target (in black) and grouped averaged values (n = 13) of CoP time series, during tracking the periodic-sinusoidal (**A**) and chaotic-Lorenz (**B**) moving target in sagittal plane on the stable (in blue) and unstable (in red) surface. The figure below provides a zoom in of the time window indicated by the vertical dashed lines.

In Fig. 2, the target signal as well as the group averaged CoP displacement on both stable and unstable surfaces are depicted, when tracking the periodic (sinusoidal) (Fig. 2A) and chaotic (Lorenz) target (Fig. 2B), respectively.

**Figure 3:**
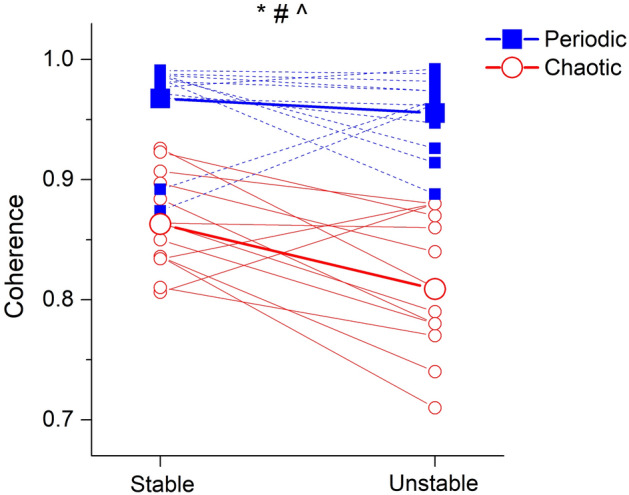
CoP-target coupling: group mean (big symbols—solid line) and individual (small symbols—dashed line) CoP-target coherence values during tracking of the periodic-sinusoidal (blue filled squares) and chaotic-Lorenz (red open circles) target motion on the stable and unstable surface. *Significant surface effect (p < 0.05); ^#^significant target complexity effect (p < 0.05); ^^^significant interaction between surface and target complexity (p < 0.05).

The CoP-target coherence values for all participants and trials ranged from 0.71 to 0.99 (Fig. 3). There was a surface (F = 5.742, p = 0.034) as well as a target complexity effect (F = 144.723, p < 0.001) on CoP-target coherence with lower values in the unstable and in the chaotic condition. Furthermore, we found an interaction (F = 6.746, p = 0.023) between surface and target complexity, indicating a greater surface-related reduction of coherence for the chaotic target tracking (Fig. 3). Post-hoc analysis confirmed that the unstable surface induced a significant decrease in CoP-target coherence only in chaotic target tracking (t(17) = 4.229, p = 0.001), but not when tracking the periodic target motion (t(14) = 0.700, p = 0.495).

### EMG activity

**Figure 4:**
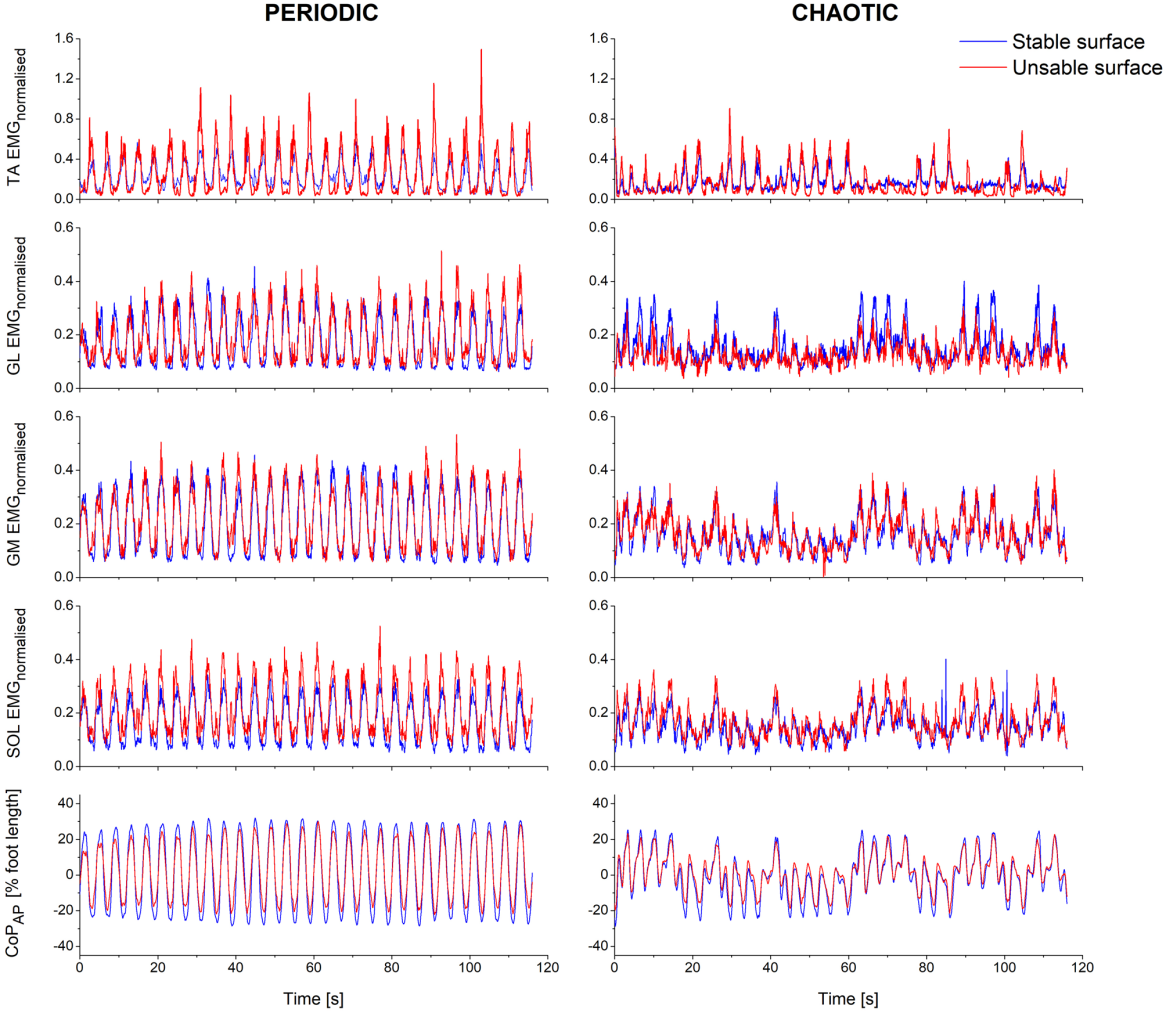
Mean curves from all participants (N = 13) per condition (periodic-sinusoidal on the left, chaotic-Lorenz on the right, Stable surface in blue and Unstable surface in red) of the normalised EMG activity of the tibialis anterior (TA), gastrocnemius lateralis (GL), gastrocnemius medialis (GM) and soleus (SOL) muscles as well as the mean curves of the anteroposterior CoP displacement.

In Fig. 4 the mean normalised EMG activity of TA, GM, GL and SOL muscles from all participants is depicted. The RMS values of the SOL and TA EMG signal were significantly greater on the unstable condition (F = 21.934, p = 0.001 and F = 15.284, p = 0.002, respectively, Table 2). However, there was no surface effect on the RMS values of the EMG signal from the GL and GM muscles (Table 2). For the chaotic target motion, we found a significant decrease of the RMS of all examined muscles, GL (F = 5.898, p = 0.032), GM (F = 9.868, p = 0.009), SOL (F = 15.282, p = 0.002) and TA (F = 39.665, p < 0.001, Table 2). Furthermore, we found for the SOL EMG activity an interaction (F = 4.823, p = 0.048) between surface and target complexity indicating, a lower surface-related increase of the SOL EMG activity for the chaotic target (Table 2). Post-hoc analysis confirmed that there was a significant surface effect on SOL EMG activity only in periodic target tracking (t(12) = -6.521, p < 0.001), but not when tracking the chaotic target motion (t(12) = -1.625, p = 0.130).

**Table 2:** EMG behaviour: RMS values of the EMG signal from the soleus (SOL_RMS_), gastrocnemius medialis (GM_RMS_), gastrocnemius lateralis (GL_RMS_) and tibialis anterior (TA_RMS_) muscles as well the ratio between TA_RMS_ and TS_RMS_ (with the TS being referred to the triceps surae muscle calculated as the average activity of GM, GL and SOL) for the four examined postural tasks of varying surface (stable-rigid and unstable-foam) and target complexity (periodic-sinusoidal and chaotic-Lorenz).

EMG activity	Stable periodic	Unstable periodic	Stable chaotic	Unstable chaotic
SOL_RMS_*^#^^	0.20 ± 0.03	0.26 ± 0.05	0.17 ± 0.07	0.20 ± 0.06
GM_RMS_^#^	0.25 ± 0.09	0.27 ± 0.08	0.19 ± 0.08	0.23 ± 0.14
GL_RMS_^#^	0.22 ± 0.13	0.25 ± 0.11	0.19 ± 0.15	0.18 ± 0.16
TA_RMS_*^#^	0.30 ± 0.08	0.44 ± 0.11	0.20 ± 0.06	0.28 ± 0.08
EMG_ratio_*^#^	1.38 ± 0.32	1.81 ± 0.43	1.19 ± 0.34	1.49 ± 0.53

Values are presented in mean ± standard deviation. *Significant surface effect (stable vs. unstable) (p < 0.05). ^#^Significant target complexity effect (periodic vs. chaotic) (p < 0.05). ^^^Significant interaction between surface and target (p < 0.05).

There was a surface (F = 6.966, p = 0.022) as well as a target effect (F = 7.543, p = 0.018) on the EMG_ratio_. On the unstable surface the EMG_ratio_ was significantly greater, while when following the chaotic target, it was significantly lower (Table 2).

## Discussion

The findings of the present study showed that (a) the surface induced perturbations increased local instability and amplified muscle coactivation and activity during postural tracking, (b) in the presence of perturbations visuo-motor coupling coherence was deteriorated, and (c) this deterioration was greater when tracking a chaotic target, implying that the effect of the induced perturbations depended on target complexity. Consequently, our both hypotheses were confirmed.

### Perturbations affect dynamic stability and CoP-target coupling

The surface-related perturbations had a significant impact on the local dynamic stability, with greater sMLE values for all kinematic data, implying a significant increase of 12% in average, in the local instability of all segments from ankle to head. Comparable increases in local instability have been reported in studies that investigated sMLE during walking and running on an even- and an uneven-surface treadmill^10^ and during a balance task on stable ground and an unstable oscillating platform^7^. Therefore, we can argue that in the present study, the tracking of the visual moving target on the unstable surface was a more demanding and more challenging task than when standing on a stable surface. On the other hand, target complexity seemed to have no significant impact on the local dynamic stability. A possible explanation for this could be the smaller sway amplitude when tracking the chaotic target, since the motion of the chaotic target did not consistently reach the amplitude of 60% foot length in every sway cycle as it was the case for the sinusoidal one.

We found that tracking performance is reduced in the presence of perturbations, as indicated by the lower coherence values in the unstable condition. In the chaotic target motion, we found a greater deterioration of tracking coherence on the unstable surface, indicating an own effect of target complexity on CoP-target coupling. We can argue that for the more complex and unpredictable target, the visuomotor process was regulated primarily by control mechanisms based on feedback information, i.e. reactive components. This may explain the higher surface-related deterioration in tracking coherence for the chaotic tracking task. The periodic sinusoidal signal is predictable compared to the chaotic one, and predictive adjustments reduce the consequence of perturbations, ensure stable motion control and improve balance performance^43–45^.

In our experimental design we used visually guided postural sway tracking tasks and we found an increase in system instability and a deterioration in tracking performance in the perturbed condition. In earlier studies we found also an increase in system instability during walking, running^10^ and continuous alternation from double-to single-leg standing^7^. Although, we cannot generalize this phenomenon in all kind of perturbations, it seems that in challenging perturbed conditions the instability is increased. There are reports that mechanical noise applied to the feet via vibrating insoles improves balance performance during quiet-stance^46,47^. However, the induced mechanical perturbations using vibrating insoles are quite minor compared to all our perturbed conditions, making the comparison of the outcomes difficult. In our opinion, the magnitude of the external mechanical noise or perturbations is a key factor that provides a trade-off between sensorimotor facilitation and increases task demand. Mechanical noise or perturbations might show a range or “sweet spot” within which promote task stability performance, with either too much or too little noise/perturbations being detrimental. Thomas et al. (2016)^48^ found also an increase in postural sway (i.e. deterioration of balance) during visually tracking moving targets in the presence of visual perturbations initiated by oscillating backgrounds depicting the destabilizing effects in postural control of demanding task conditions. Similarly, Oullier et al.^26^ creating optic flow in postural control using the so-called moving room paradigm found a deterioration of the visual coupling between room and head motion as well as the ankle-hip coordination with increasing the oscillation frequency of the moving room evidencing the effect of task demand on postural coordination.

Summarising the above, we can argue that for the complex target task the sensory information, i.e. vision and somatosensory, seemed to play a more important role in the movement control loop, compared to the less complex task. It is possible that in the case of the complex task, the perturbations-induced fluctuation in the sensorimotor system affected multisensory integration to a greater extent, and thus led to greater deterioration in task performance.

### Neuromuscular responses in presence of perturbations

The postural tracking on the unstable surface introduced in our case repeated and variable perturbations which increased the instability of the human body and challenged the motor control of the tasks. As a consequence, the EMG activity of both SOL and TA increased on the unstable surface in both investigated postural tasks. Furthermore, the EMG_ratio_ also increased in the unstable condition, indicating a higher coactivation of the muscles acting at the ankle joint, and particularly greater contribution of the TA for the task regulation and control. The found differences in the muscle activation patterns evidenced modifications of motor control in the unstable condition, which might make motor execution less prone to the influence of disturbances. All participants were able to track the periodic as well as the chaotic target motion regardless the presence of perturbations, as indicated by the coherence values. This implies that the motor system was able to deal with the perturbations, indicating a robust motor control. On the other hand, the participants demonstrated a declined accuracy in the tracking of the target cues on the unstable surface.

Typically, the role of the SOL muscle together with other plantarflexor muscles during postural sway is to stop and counteract the anterior body motion, while the TA muscle together with other dorsiflexors muscles are mainly responsible for regulating and blocking the posterior motion^49^. A higher activation of these muscles on the unstable surface may be a compensating mechanism to the induced perturbations and may explain the system’s ability to maintain its functionality in CoP-target coupling. We found such upregulation in the EMG activity of the SOL and TA muscles on the unstable surface, but no effects were found on GM and GL. This inconsistency in the activation patterns between the plantarflexor muscles may be explained by the different way that these are involved in postural control. During standing, SOL is more actively modulated as an active agonist, while the gastrocnemii have also periods of un-modulated activity^50^. Taking additionally into account that the SOL muscle has the largest physiological cross-sectional area compared to gastrocnemius medialis and lateralis (62 ± 5%, 26 ± 3% and 12 ± 2% of the entire TS physiological cross sectional area, respectively)^51^, i.e. it is the strongest muscle of the TS as well as it consists mainly of type I muscle fibres (slow twitch)^52^, it becomes evident that the SOL muscle was the most important one of the TS for postural control during the examined swaying task.

Summarising the above, we found that the activation patterns were altered on the unstable surface, and this alteration was depending on target complexity. While there was a modulation of the neuromotor system towards amplification of muscle coactivation and activity in order to compensate the surface-related perturbations and to ensure robust motor control, this modulation was smaller when tracking the chaotic target, indicating a more conservative neuromuscular modulation in the chaotic, more complex task.

A limitation of the study is that the maximum amplitude of the tracking task was set at 60% of the foot length. It is possible that greater amplitudes may have provoked a more challenging task. Nevertheless, we found a similar increase in the instability on the unstable surface compared to the rigid one, to previous studies^7,10^, indicating that the amplitude and the oscillation frequency used at the present study was adequate.

## Conclusions

We found that external perturbations, induced by unstable surface, increased local instability and deteriorated CoP-target coupling during postural tracking tasks. Visuo-motor deterioration is greater when tracking a chaotic compared to a sinusoidal target, implying that the effect of the induced perturbations depends on target complexity. The neuromotor system modulates its responses towards amplification of muscle coactivation and activity in order to compensate the surface-related perturbations and to ensure robust motor control.
